# Thermoresponsive Poly(ε-Caprolactone)-Poly(Ethylene/Propylene Glycol) Copolymers as Injectable Hydrogels for Cell Therapies

**DOI:** 10.3390/polym12020367

**Published:** 2020-02-07

**Authors:** Kyle Brewer, Batjargal Gundsambuu, Paula Facal Marina, Simon C. Barry, Anton Blencowe

**Affiliations:** 1 Applied Chemistry and Translational Biomaterials (ACTB) Group, School of Pharmacy and Medical Sciences, University of South Australia, Adelaide, South Australia 5000, Australia; kyle.brewer@mymail.unisa.edu.au (K.B.); Paula.FacalMarina@unisa.edu.au (P.F.M.); 2 Cooperative Research Centre for Cell Therapy Manufacturing, University of South Australia, Adelaide, South Australia 5000, Australia; batjargal.gundsambuu@adelaide.edu.au (B.G.); simon.barry@adelaide.edu.au (S.C.B.); 3 Molecular Immunology, Robinson Research Institute, University of Adelaide, Adelaide, South Australia 5005, Australia; 4 Future Industries Institute, University of South Australia, Mawson Lakes, South Australia 5095, Australia; 5 Department of Gastroenterology, Women’s and Children’s Hospital, SA Health, Adelaide, South Australia 5006, Australia

**Keywords:** thermoresponsive, hydrogel, poly(ethylene glycol), poly(propylene glycol), polycaprolactone, adoptive cell therapy, ACT, injectable, delivery, gelation, LCST

## Abstract

Injectable, thermoresponsive hydrogels are promising candidates for the delivery, maintenance and controlled release of adoptive cell therapies. Therefore, there is significant interest in the development of cytocompatible and biodegradable thermoresponsive hydrogels with appropriate gelling characteristics. Towards this end, a series of thermoresponsive copolymers consisting of poly(caprolactone) (PCL), poly(ethylene glycol) (PEG) and poly(propylene glycol) (PPG) segments, with various PEG:PPG ratios, were synthesised via ring-opening polymerisation (ROP) of ε-caprolactone and epoxy-functionalised PEG and PPG derivatives. The resultant PCL–PEG–PPG copolymers were characterised via proton nuclear magnetic resonance (1H NMR) spectroscopy, gel permeation chromatography (GPC) and differential scanning calorimetry (DSC). The thermoresponsive characteristics of the aqueous copolymer solutions at various concentrations was investigated using the inversion method. Whilst all of the copolymers displayed thermoresponsive properties, the copolymer with a ratio of 1:2 PEG:PPG exhibited an appropriate sol–gel transition (28 °C) at a relatively low concentration (10 wt%), and remained a gel at 37 °C. Furthermore, the copolymers were shown to be enzymatically degradable in the presence of lipases and could be used for the encapsulation of CD4+ T-cell lymphocytes. These results demonstrate that the thermoresponsive PCL–PEG–PPG hydrogels may be suitable for use as an adoptive cell therapy (ACT) delivery vehicle.

## 1. Introduction

A substantial amount of research has been conducted into the design and synthesis of thermoresponsive hydrogels for use as cell culture surfaces [1,2,3], 3D bioprinting matrices [4] and controlled-release drug delivery systems [5,6]. Many of these systems rely upon hydrogels exhibiting a lower critical solution temperature (LCST), whereby gelation is induced by a positive temperature differential [7,8,9], and the gel state facilitates cell proliferation (e.g., 3D bioprinting matrices) or drug release. The extracellular matrix-like biocompatibility of thermoresponsive hydrogels is essential for many applications, especially tissue engineering, wherein the proliferation of cells within the hydrogel construct is crucial for producing viable tissues.

However, one of the most clinically promising applications of thermoresponsive hydrogels, is their potential use in adoptive cell therapy (ACT). Generally, treating cancer with ACT involves the isolation, expansion and infusion of a patient’s lymphocytes (commonly T-cell lymphocytes). The isolated lymphocytes may innately recognise and target cancerous tumours or be modified ex vivo to do so, and upon infusion destroy cancerous material, in a manner akin to an immune response [10,11,12,13]. Currently, lymphocytes are administered intravenously and whilst this makes ACT effective against blood-borne cancers such as Acute Lymphoblastic Leukaemia [12], it is particularly ineffective against neoplastic cancers, due in part to the inability to prevent the diffusion of lymphocytes away from the target site, and compounded by Cytokine Release Syndrome (CRS). Invariably, the effectiveness of a cancer treatment can be improved via its localisation at the tumour site, which in turn can greatly reduce its harmful side effects. By localising the lymphocytes at the target site and restricting their movement, the number of lymphocytes required to ensure adequate exposure to the cancerous material may be reduced, as well as the risk of CRS. As such, thermoresponsive hydrogels exhibit the potential to increase the efficacy of ACT, whilst simultaneously reducing related side effects.

For a thermoresponsive hydrogel to be appropriate for ACT, it is required to gel at physiological temperature (37 °C), be injectable, cyto/biocompatible, and biodegradable, whilst having its biocompatibility retained by its degradation products. Further, ensuring the hydrogel and its degradation products are cyto/biocompatible, enables a lymphocyte-loaded hydrogel to degrade over time and be excreted or metabolised. This negates the need for complex follow-up procedures to remove non-biodegraded material, or to be constrained to the use of low molecular weight polymers (to facilitate renal excretion). Recent research has produced very few thermoresponsive hydrogels that satisfy the entirety of these requirements. At present, research conducted by Monette et al. [14] and Tsao et al. [12] appear to be the pioneering works for this application. Both groups investigated the preparation of thermoresponsive hydrogels based upon chitosan, a naturally occurring polysaccharide obtained from the exoskeletons of arthropods. The poly(ethylene glycol)-grafted chitosan (PEG-chitosan) hydrogel prepared by Tsao et al. and the chitosan hydrogel (with NaHCO_3_ and phosphate buffer gelation agents) prepared by Monette et al. were both found to exhibit the appropriate thermoresponsive behaviour. They were also determined to be cytocompatible with T-cell lymphocytes. However, this does not necessarily indicate the in vivo biocompatibility and biodegradation of the hydrogels. As outlined by Kean and Thanou [15], the toxicity and biodegradability of chitosan-based materials remain a source of contention, and further research may reveal the incompatibility of these hydrogels with in vivo applications. This caveat can be circumvented by utilising monomers that already possess known in vivo compatibility.

Pluronics (also known as Poloxamers) are synthetic block copolymers consisting of PEG and PPG in linear PEG–PPG–PEG sequences. Pluronics are injectable, thermoresponsive hydrogels that exhibit an LCST and are well known for their biocompatibility [16] and tuneable sol–gel temperatures (through modification). These properties have seen Pluronic-based formulations extensively investigated for their suitability for tissue engineering [17,18], drug [19,20,21] and cell delivery [22,23], as facilitated by in-situ triggering of the sol–gel transition. However, Pluronic hydrogels exhibit low mechanical strength and undergo rapid dissolution in an aqueous medium, which may impede the sustained in vivo release of cells for ACT [24,25,26]. These issues are exacerbated by the high concentrations of Pluronics required in solution (>15 wt%) [22,26,27] to achieve robust gelation at physiological temperature, which reduces the degree to which Pluronic formulations can be impregnated with cells and may also impede cell migration due to the high density of the gel constructs afforded by high polymer concentrations. Further, despite their excellent biocompatibility, Pluronics are not biodegradable [16,24] without the introduction of biodegradable moieties. For ACT, the degradation of the gel matrix is of great importance, as it can affect the rate and extent to which cells are released from the matrix—either through degradation itself, or migration through the matrix as it undergoes degradation.

It is through the modification of traditional Pluronics via copolymerisation, that these shortcomings can be overcome. For example, the copolymerisation of Pluronic F127 with hexamethylene diisocyanate (HDI), saw a 15-fold increase in the viscosity of the resultant copolymer at physiological temperature, and the presence of a sol–gel transition at concentrations as low as 5 wt% [28]. Therefore, in an attempt to further improve the characteristics of Pluronics for application in ACTs, we hypothesised that the grafting of PEG and PPG blocks to a biodegradable poly(caprolactone) (PCL) backbone would provide thermoresponsive copolymers with enhanced enzymatic degradability. Furthermore, PCL-based copolymers offer a simple, cost-effective synthesis method, via one-pot ring-opening polymerisation (ROP) [29], potentially enabling the large-scale production of the copolymers for clinical and commercial use. Herein, we report the synthesis and characterisation of thermoresponsive PCL–PEG–PPG copolymers and conduct a preliminary assessment of their suitability for encapsulating lymphocytes.

## 2. Materials and Methods

### 2.1. Materials

All reagents were used as received unless otherwise specified. Poly(ethylene glycol) diglycidyl ether (EPEG, *M*_n_ = 500 Da), poly(ethylene glycol) monomethyl ether (MPEG, *M*_n_ = 750 Da), poly(propylene glycol) diglycidyl ether (EPPG, *M*_n_ = 640 Da), ε-caprolactone (CL, 97%), stannous octoate (tin(II) 2-ethylhexanoate; 92.5–100%), and lipases from *Candida rugosa* and *Rhizopus oryzae* were obtained from Sigma Aldrich. Analytical reagent grade hexanes and tetrahydrofuran (THF) were purchased from Chem-Supply. Toluene (≥99.8%) was purchased from RCI Labscan. Deuterium oxide (D_2_O) and deuterated chloroform (CDCl_3_) were purchased from Cambridge Laboratory Isotopes. Polystyrene standards for gel permeation chromatography (GPC) were purchased from Fluka. Phosphate buffered saline (PBS) tablets were purchased from Sigma Aldrich and PBS solutions were prepared following the manufacturer’s guidelines. High-purity water with a resistivity of greater than 18 MΩ.cm was obtained from an in-line Millipore RiOs water purification system. 

Recombinant Human CCL17/TARC Protein (CCL17) and MIP-3α (CCL20) were purchased from RayBiotech. RosetteSep Human CD4+ T-cell Enrichment Cocktail was obtained from Stem Cell Technologies (Vancouver, BC, Canada). HyClone Dulbecco’s PBS (Hyclone PBS) and Ficoll-Paque Plus (Ficoll) were purchased from GE Healthcare Life Sciences. Fetal Bovine Serum (FBS) and 4-(2-hydroxyethyl)-1-piperazineethanesulfonic acid (HEPES) were obtained from Gibco (Waltham, MA, USA). X-VIVO 15 was purchased from Lonza (Basel, Switzerland). Human Serum and L-Glutamine were purchased from Sigma (St. Louis, MI, USA). IL-2/Proleukin was obtained from Novartis Vaccines and Diagnostics (Basel, Switzerland). Carboxyfluorescein succinimidyl ester (CFSE) was purchased from Affymetrix eBioscience (Waltham, MA, USA).

### 2.2. Instrumentation

Proton nuclear magnetic resonance (^1^H NMR) spectroscopy was conducted on a Bruker Avance II 300 MHz spectrometer (Billerica, MA, USA), using the solvent signal as an internal reference. The composition of the copolymers was calculated using the ratios of the integral values of characteristic resonances at δ_H_ 4.0 (methylene group of PCL repeat unit), 3.6 (methylene group of PEG repeat unit), 1.1 (methyl group of PPG repeat unit) and 2.8 (methine group of epoxide end-groups) ppm.

Gel permeation chromatography (GPC) was performed on a Viscotek GPCmax (VE 2001, Houston, TX, USA) system using two PLgel Mixed-C columns (Agilent) in series and operating at 40 °C. THF was used as the eluent at a flow rate of 1 mL.min^−1^. OmniSEC 5.12 software (Malvern Instruments, Malvern, UK) was used to determine molecular weight characteristics based upon a conventional column calibration with narrow molecular weight polystyrene standards. 

Differential scanning calorimetry (DSC) was performed on a Discovery DSC (TA Instruments, New Castle, DE, USA) under a nitrogen atmosphere. The copolymers and pure PCL were heated from 25 to 100 °C, cooled to −80 °C and then heated again to 100 °C before finally being cooled to 25 °C. All heating and cooling cycles were conducted at a ramp rate of 10 °C.min^−1^.

### 2.3. Polymer Synthesis

#### 2.3.1. Synthesis of PCL–PEG Copolymer

The PCL–PEG copolymer was synthesised via one-pot ring-opening polymerisation using CL (10.3 g, 97.4 mmol) and EPEG (12.3 g, 24.6 mmol) as (macro)monomers, and MPEG (2.16 g, 2.88 mmol) as an initiator, according to the method described by Lin et al. [29] The reagents were dissolved in a RB flask under nitrogen and stannous octoate (0.180 g, 0.4 mmol) was added. After stirring at 120 °C for 20 h the copolymer was precipitated from hexanes (500 mL) and isolated via centrifugation. The precipitate was then dried under vacuum (0.1 mbar, 25 °C) to afford the copolymer as a waxy solid. ^1^H NMR (CDCl_3_, 300 MHz) δ_H_ 4.01 (*t*, C**H_2_**O, PCL), 3.59 (*s*, C**H_2_**O, PEG), 3.13–3.08 (*m*, C**H**O, epoxide end-group), 2.76–2.72 (*m*, C**H**O, epoxide end-group), 2.57–2.54 (*m*, C**H**O, epoxide end-group), 2.26 (*t*, C**H_2_**CO, PCL), 1.65–1.55 (*m*, C**H_2_**CH_2,_ PCL), 1.39–1.28 (*m*, CH_2_C**H_2_**, PCL) ppm.

#### 2.3.2. Synthesis of PCL–PEG–PPG Copolymers

PCL–PEG–PPG copolymers were synthesised according to the method described for the PCL–PEG copolymer, with the addition of EPPG. A series of PCL–PEG–PPG copolymers were prepared by systematically varying the EPEG:EPPG mole ratio to allow the effects of these changes on the copolymers’ properties to be investigated. These polymers were denoted as PEG*_x_*PPG*_y_*, where *x* and *y* represent the mole ratio of the EPEG and EPPG monomers used in their synthesis, respectively. The ratios of the reagents used for each copolymer are provided in Table 1, and the quantities of the reagents are provided in the Supporting Information (SI), Table S2. In all cases, the amount of CL (11.1 g, 97.5 mmol) and anhydrous toluene (5 mL) remained constant, as well as the mole ratio of the catalyst stannous octoate to the MPEG initiator (1:6.8). For all PEG_x_PPG_y_ copolymers a 1:34 mole ratio of MPEG:CL was employed, with the exception of M_0.7_PEG_1_PPG_2_, which employed a 1:48 mole ratio. The copolymers were isolated as waxy solids in yields of 66–75%. All of the PEG_x_PPG_y_ copolymers displayed similar ^1^H NMR spectra: ^1^H NMR (CDCl_3_, 300 MHz) δ_H_ 4.05 (*t*, C**H_2_**O, PCL), 3.63 (*s*, C**H_2_**O, PEG), 3.80–3.36 (*br m*, C**H_2_** and C**H**, PPG), 3.17–3.10 (*m*, C**H**O, epoxide end-group), 2.80–2.75 (*m*, C**H**O, epoxide end-group), 2.63–2.58 (*m*, C**H**O, epoxide end-group), 2.30 (*m*, C**H_2_**CO, PCL), 1.69–1.58 (*m*, C**H_2_**-CH_2,_ PCL), 1.42–1.32 (*m*, CH_2_C**H_2_**, PCL), 1.13 (*d*, C**H_3_**CH_,_ PPG) ppm.

### 2.4. Characterisation

#### 2.4.1. Sol–gel Profiles

Sol–gel profiles were constructed for the copolymers at known wt% concentrations. Solutions of 5–40 wt% copolymer were prepared in PBS by initially melting the copolymer at 50 °C and then adding the required amount of PBS at 50 °C. The mixtures were vortex mixed to homogeneity and then cooled to 4 °C for 5 min. The solutions were then heated to 52 °C in 4 °C increments, with each increment sustained for 2 min to allow the solutions to equilibrate. Following equilibration at each temperature increment, the phase state of the solutions was determined through qualitative observation using the inversion method, as outlined in previous studies [29,30,31,32].

#### 2.4.2. Injectability Testing

Injectability tests were performed on a Shimadzu EZ-LX Universal Tester fitted with a 50 N load cell and compression platen. The compression platen was placed in contact with the syringe plunger, using a custom 3D-printed platform that clamped the syringe and oriented the syringe needle downwards. Testing was conducted at a constant stroke rate of 1 mm/s to represent manual syringe operation, until the plunger contacted the end of the syringe barrel. Each of the formulations were tested six times and the results averaged. All tests were conducted at ambient temperature (≈23 °C). Disposable 1 mL Soft-Ject Luer Slip syringes (Henke Sass Wolf) fitted with a 25 G (0.50 × 19 mm) needle tip (Neolus) were used for all tests, with a sample volume of 1 mL extruded directly into air during testing. The dynamic glide force (DGF) was calculated within the “plateau” region as previously reported [33], by calculating the average force measured across a displacement of 5–35 mm, for all six measurements. The plateau region was determined to be within this range as it excluded variations in measured force due to the approach of the plunger to the end of the syringe barrel. Similarly, the maximum force required during injection (F_max_) was determined as the average maximum force measured within the plateau region. The plunger-stopper break loose force (PBF) was determined as the average maximum force within the bounds of the PBF peak (approximately 0.15 mm displacement).

#### 2.4.3. Enzyme Degradation Study

The 5 wt% mixtures of PEG_1_PPG_2_ in D_2_O were prepared by melting the copolymer at 50 °C for 5 min and then adding the required amount of D_2_O. The mixtures were vortex mixed to homogeneity. Subsequently, lipases from *Candida rugosa* and *Rhizopus oryzae* were added with vortex mixing to afford a lipase concentration of 10 mg·mL^−1^, and the mixtures were incubated at 37 °C for 48 h. The mixtures were separated into two phases via centrifugation and the D_2_O supernatant was collected and analysed via ^1^H NMR spectroscopy. The insoluble component was collected and dried under vacuum (0.1 mbar, 25 °C) prior to ^1^H NMR spectroscopic analysis in CDCl_3_.

#### 2.4.4. CD4+ T-Cell Lymphocyte Isolation and Culture

Fresh buffy coat was transferred into a flask with Rosettesep Human CD4+ T-cell Enrichment Cocktail at 1 mL per 50 mL of buffy coat, and incubated on an orbital mixer (300 rpm) for 20 min at 22 °C. The blood sample was diluted with a solution of Hyclone PBS and 2% FBS (1:2) and gently mixed. A 50 mL centrifuge tube was then charged with 15 mL of Ficoll, and 35 mL of diluted blood was layered upon the Ficoll. The resultant mixture was then centrifuged for 25 min at 400× *g* (22 °C, brake off). From the plasma interphase, the enriched CD4+ T-cells were collected into a separate tube and washed twice with the Hyclone PBS and 2% FBS (1:2) solution. Following each wash, the cells were pelletised by centrifugation at 1200 rpm for 5 min (22 °C). The isolated CD4+ T-cells were maintained in cell medium prepared by supplementing X-Vivo 15 Complete Medium with 5% human serum, 20 nM HEPES and 2 mM L-glutamine plus IL-2 (500 U·mL^−1^). 

#### 2.4.5. Fluorescent-Labelling of T-Cell Lymphocytes

A single cell suspension of the cells was prepared and washed twice with Hyclone PBS to remove any remaining serum. The cells were then resuspended in pre-warmed (22 °C) Hyclone PBS at a density of 5 × 10^6^ cells·mL^−1^. CFSE was then added and mixed to afford a concentration of 15 μM, and the mixture was incubated whilst covered for 10 min at 22 °C. The labelling was then stopped through the addition of 4–5 volumes of cold cell medium and incubation on ice for 5 min. Finally, the cells were washed three times with fresh cell medium.

#### 2.4.6. Cell Encapsulation and Survival

The 20 and 30 wt% stock solutions of PEG_1_PPG_2,_ were prepared by melting the copolymer at 50 °C for 5 min and then dispersing the melted copolymer in the appropriate amount of cell media via vortex mixing. 50 μL aliquots of the stock solutions were transferred to transwell plate inserts (Corning, NY, USA) followed by the immediate addition of 50 μL of concentrated cell suspension (CCS), thus simultaneously diluting the stock solutions to the desired polymer wt% and introducing CD4+ T-cell lymphocytes; the 30 wt% solution was diluted to 15 wt% to provide a final seeding density of 1 × 10^6^ cells and the 20 wt% solutions were diluted to 10 wt% to provide seeding densities of 1 × 10^6^ and 5 × 10^6^ cells, respectively. Controls were prepared by transferring 50 μL of cell medium and 50 μL of CCS to afford a seeding density of 1 × 10^6^ cells. All copolymer and control solutions were then incubated for 24 h at 37 °C, resulting in the gelation of both 10 wt% solutions and the 15 wt% solution. The transwell plate was incubated at 37 °C for 24 h. After the 24 h incubation period, qualitative observations of the Transwell insert were made using fluorescence microscopy, to determine the encapsulation and viability of the cells.

## 3. Results and Discussion

**Scheme 1:**
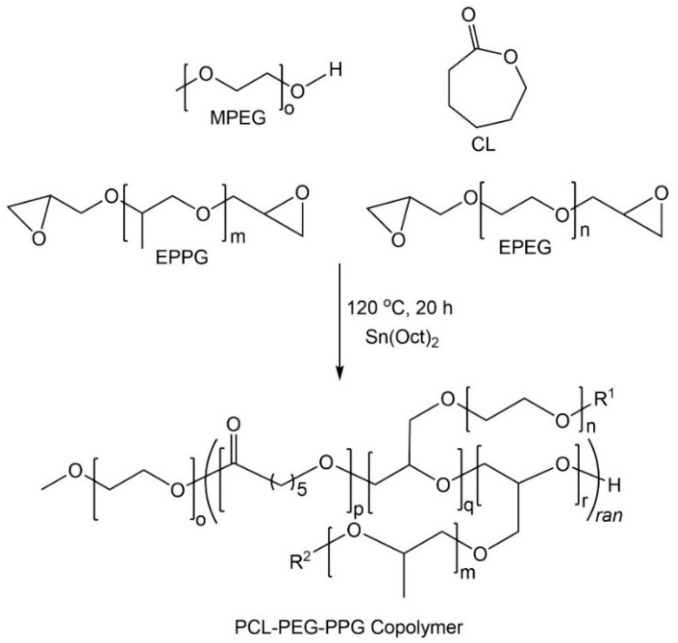
Synthesis of poly(ε-caprolactone)-poly(ethylene/propylene glycol) (PCL–PEG–PPG) copolymers via ring-opening polymerisation (ROP).

The poly(ε-caprolactone)-poly(ethylene/propylene glycol) copolymers (PCL–PEG–PPG) were synthesized via the ROP of CL, EPEG and EPPG, using MPEG as the initiator and stannous octoate as the catalyst (Scheme 1) [29]. This approach results in branched copolymers consisting of polyester-ether backbones derived from the ring-opening of CL and the epoxide groups (resulting in glycol repeat units), and pendant or cross-linking PEG and PPG chains. The mole ratio of EPEG to EPPG was varied to produce a series of copolymers (Table 1) with different thermoresponsive characteristics, whilst the CL to total EPEG and EPPG mole ratio was kept constant at ~1:0.26 (repeat unit (RU) ratio ~1:2) and the MPEG:CL mole ratio was 1:34 (PEG_x_PPG_y_). In addition, a copolymer was prepared with a reduced MPEG:CL mole ratio (1:48) (M_0.7_PEG_1_PPG_2_). The copolymers were denoted as PEG_x_PPG_y_, where *x* and *y*, represent the mole ratio of the EPEG and EPPG macromonomers used in their synthesis, respectively.

### 3.1. Copolymer Characterisation

**Figure 1:**
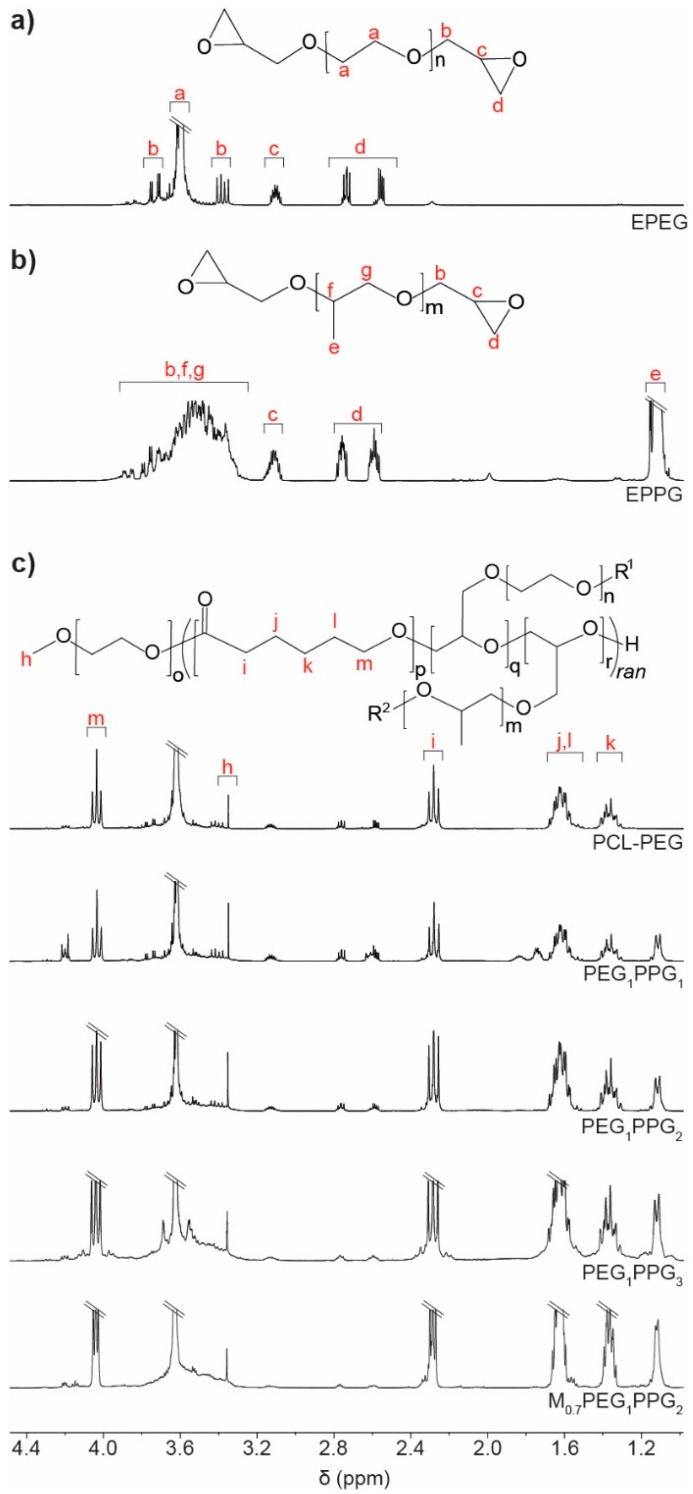
^1^H NMR spectra (CDCl_3_) of the macromonomers (**a**) EPEG and (**b**) EPPG, and (**c**) PCL–PEG and PCL–PEG–PPG copolymers: PEG_x_PPG_y_, where *x* and *y*, represent the mole ratio of the EPEG and EPPG macromonomers used in their synthesis.

^1^H NMR spectroscopic analysis was used to characterise the copolymers and determine their composition. Comparison with the EPEG and EPPG monomer spectra (Figure 1a) revealed that the copolymers contained both PEG and PPG components (Figure 1b), with characteristic resonances at ~3.6 and 1.1 ppm corresponding to the backbone methylene and methyl protons of the PEG and PPG chains, respectively. The inclusion of PCL into the copolymer structure was evident from a characteristic resonance at ~4 ppm corresponding to the methylene group adjacent to the ester linkage, as well as other typical resonances at ~2.3, 1.6 and 1.4 ppm [30].

To determine the composition of the copolymers, the integral ratios of the PCL, PEG and PPG repeat units were calculated from the characteristic resonances at ~4.0, 3.6 and 1.1 ppm, respectively, and used to determine the mole ratio of those components (Table 1). For PCL–PEG, the mole ratio of monomer components present in the copolymer were approximately the same as the initial monomer feed mole ratio. However, for the PEG_x_PPG_y_ copolymers a clear reduction in the amount of PEG and PPG relative to PCL was noted in the copolymers when compared to the initial monomer feed mole ratio, implying incomplete incorporation of the EPEG and EPPG monomers into the copolymers. In particular, EPPG appeared to be incorporated to a lesser extent, although the reason for this is unclear given that both EPEG and EPPG have similar molecular weights and identical reactive epoxide functionalities. Regardless, the ratio of PPG relative to PEG in the copolymers was found to increase with increasing EPPG in the monomer feed ratio. 

As the EPEG and EPPG components can be incorporated into the PCL chains in several different sequences, a number of different resonances may result from each. For instance, a glycerol repeat unit derived from a ring-opened epoxide may reside between two CL repeat units, or glycerol units may be adjacent to one another. The resonance at 4.2 ppm in the copolymer spectra is consistent with a glycerol methine adjacent to an ester functional group, which indicates the presence a CL-glycerol-CL repeat unit sequence in the copolymer structure [34]. The presence of the glycerol repeat unit is evidence of the ring-opening and incorporation of the epoxide end-groups of EPEG and EPPG into the copolymers.

In all of the copolymers, it was noted that some epoxide groups remained (resonances at 2.6–3.1 ppm, Figure 1b), which may result from pendant chains in the copolymers or unincorporated EPEG and EPPG monomers (vide infra). However, in addition to the presence of resonances from glycerol repeat units, the incorporation of the EPEG and EPPG monomers into the copolymer structure was also confirmed by the reduction in the number of epoxide end-groups. Using the known amounts of PEG and PPG calculated from the copolymer spectra, it is possible to determine the theoretical epoxide end-group integral values if the respective monomers were unincorporated in the copolymer, which were compared to the actual epoxide integral values. In all cases, there was a reduction in the epoxide integral values, which corresponded to a degree of epoxide incorporation into the copolymers of 23%–54% (SI, Table S1). 

The presence of glycerol repeat units and the reduction of epoxide resonances in the NMR spectra of the copolymers are a good indication that the EPEG and EPPG monomers are incorporated into the copolymer structure. In addition, the presence of pendant epoxide groups in the copolymers can be highly advantageous as they allow further derivatisation, which could be used to tune the copolymers’ properties or incorporate functionality for specific applications (e.g., peptides/proteins for cellular interactions and signalling).

**Figure 2:**
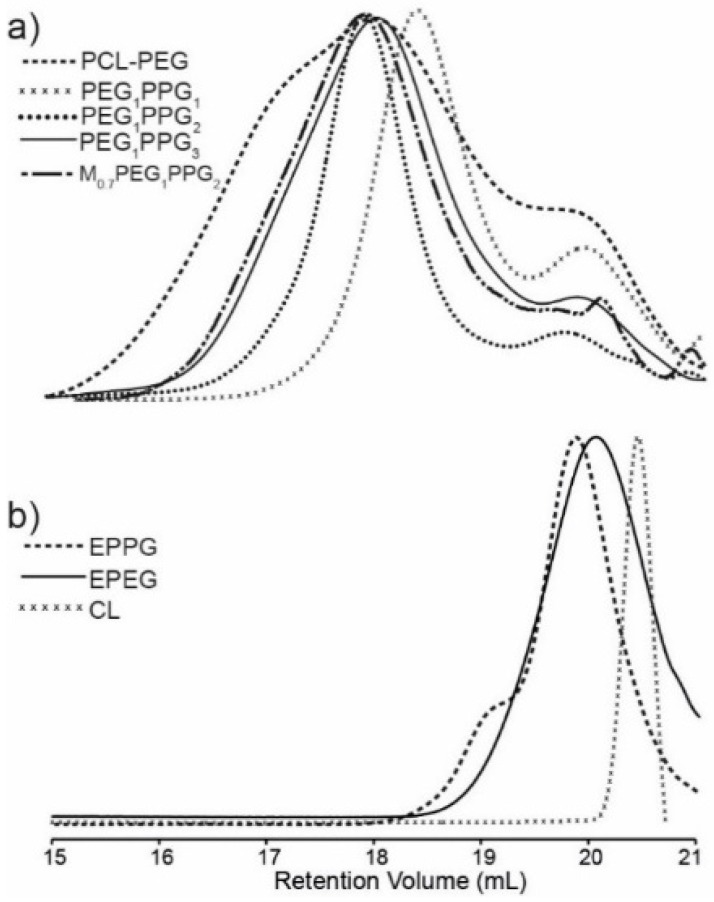
GPC differential refractive index (DRI) chromatograms of (**a**) PCL–PEG and PEG_x_PPG_y_ copolymers and (**b**) EPPG, EPEG and CL macromonomers.

Gel permeation chromatography (GPC) of the copolymers revealed broad multimodal distributions (Figure 2a), with smaller peaks towards higher retention volume (~20 mL), consistent with the presence of unincorporated EPEG and EPPG monomers (Figure 2b). In combination, the NMR spectroscopy and GPC results indicate that the copolymer mixtures contain EPEG and EPPG that have been incorporated into the copolymer structure and a small proportion of unincorporated macromonomers that are not completely removed during the copolymer isolation. GPC analysis of the copolymers provided weight-average molecular weight (*M*_w_) values ranging from 2.0–5.9 kDa and broad dispersity values (*Ð* > 2), which result from branching of copolymer chains and the presence of unincorporated low molecular weight EPEG and EPPG macromonomers. The *M*_w_ of the PCL–PEG copolymer is consistent with previous reports [29].

**Figure 3:**
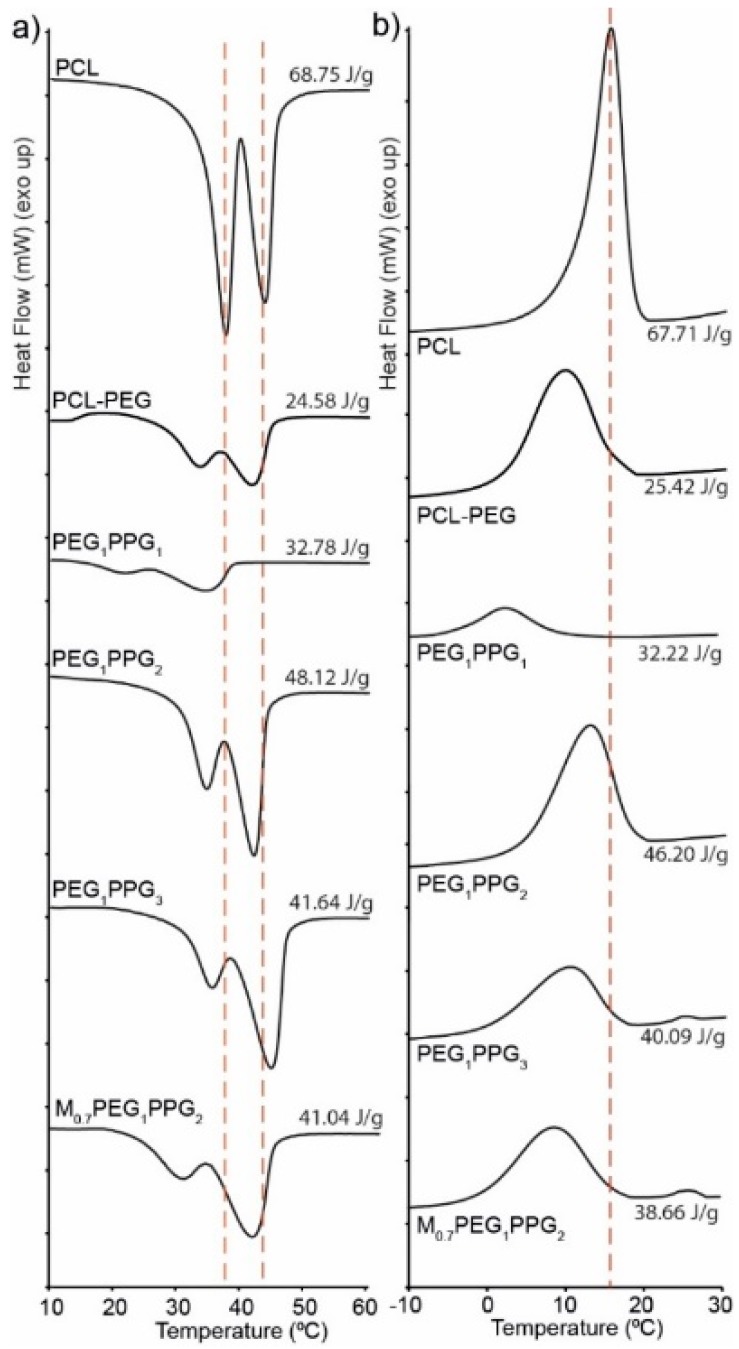
DSC thermograms of a PCL homopolymer and the copolymers showing (**a**) melting peaks and (**b**) crystallising peaks obtained on the second heating and cooling cycles, respectively.

The thermal transitions of the copolymers were assessed via differential scanning calorimetry (DSC) and compared with that of a PCL homopolymer (Figure 3). The copolymer thermograms exhibit similar features to that of pure PCL with some notable exceptions. For pure PCL, there exists two overlapping endothermic melting transitions (*T_m_*) at ~38 and 43 °C, corresponding to two different crystallite regions (Figure 3a). Whilst these melting transitions are also observed in the copolymers, they are in the most part shifted to lower temperatures, with a more pronounced shift and reduction in intensity being noted for the lower temperature transition [35]. The observed differences indicate that the incorporation of PEG and PPG branches into the copolymers interferes with the crystal packing of the PCL segments, resulting in smaller crystallites. This is also supported by the exothermic crystallisation transition (*T_c_*), which broadens and shifts to lower temperatures (~10 °C) for the copolymers as compared to the PCL homopolymer (~17 °C), indicating that the PEG and PPG branches hinder crystallisation of the PCL segments. It should be noted that no thermal transitions resulting from the PEG or PPG components were observed in the DSC thermograms, most likely as a result of their short block lengths and segregation within the PCL matrix.

### 3.2. Copolymer Gelation Studies

**Figure 4:**
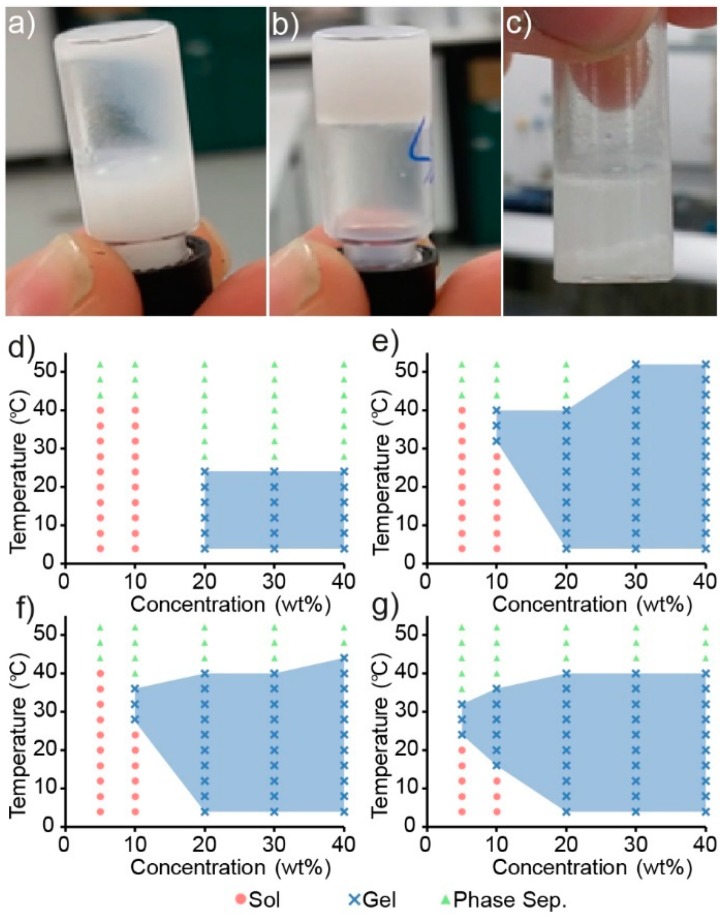
Digital images of PEG_1_PPG_3_ when present in (**a**) solution (10 wt%, 4 °C) and (**b**) gel (10 wt%, 37 °C) states, and as a (**c**) phase separated mixture (10 wt%, 50 °C). Sol–gel phase diagrams for (**d**) PEG_1_PPG_1_, (**e**) PEG_1_PPG_2_, (**f**) PEG_1_PPG_3_ and (**g**) M_0.7_PEG_1_PPG_2_ copolymers, with coloured symbols denoting the observed phase state of the solutions when inverted, and blue shaded areas denoting the observed gel regions.

Sol–gel phase diagrams for the copolymers were constructed using the inversion method [29,31,36], whereby a vial containing the polymer solution was gradually heated and inverted at predetermined temperatures. If the solution was observed to flow it was deemed a solution (Figure 4a), and if no flowing was observed it was deemed a gel (Figure 4b). Furthermore, if the copolymer was observed to exclude the solution, forming two distinct layers, it was labelled a phase separated mixture (Figure 4c).

Copolymer mixtures of various concentrations (wt%) were prepared in PBS and heated in 4 °C increments from 4 to 52 °C with a 2 min equilibrium period at each temperature before inversion. The copolymers were found to be poorly soluble in aqueous solutions, and careful mixing was required to ensure a homogenous dispersion. Heating of the copolymer to form a melt prior to the addition of PBS proved to be the most reliable method, whereas attempts to directly dissolve the solid polymer in PBS resulted in heterogeneous mixtures that would either fail to undergo a sol–gel transition or produce inconsistent results.

Interestingly, the gelation of the PCL–PEG copolymer was not observed across the temperature range tested, even at high polymer concentrations, which is contrary to that reported previously [29]. Whilst the formation of a precipitate was observed in the PCL–PEG solution upon heating, gross gel formation was absent. Sol–gel phase diagrams of the PEG and PPG containing copolymers are shown in Figure 4d–g and demonstrate that by copolymerising both PEG and PPG with PCL (as opposed to solely PEG and PCL), a thermoresponsive copolymer was obtained. Furthermore, increasing the amount of PPG in the copolymer appeared to increase its thermoresponsiveness (Figure 4f,g), whereby the gel-forming temperature range is increased, and supports the hypothesis that the incorporation of thermoresponsive PPG moieties would impart a greater thermoresponsiveness to the copolymers. These results are consistent with the extensively studied linear PEG–PPG–PEG polymers (also known as poloxamers, or by their trade names, Pluronics), which demonstrate similar thermoresponsive characteristics.

Although the exact mechanism is currently disputed, the gelation of poloxamers may be explained by the formation of micelles and self-assemblies as a function of temperature and to a lesser extent, concentration [37]. Both the PEG and PPG blocks are hydrated below a specific temperature, at which point the PPG blocks are soluble in solution, despite their decreased hydrophilicity relative to PEG. Upon an increase in temperature to the critical micelle temperature (CMT) the PPG blocks experience a reduction in solubility which drives the formation of micelles and aggregates, which are the driving force behind gelation. Furthermore, the proportion of PPG in poloxamers controls both the critical micelle concentration (CMC) and the CMT [38]. For example, at constant concentrations, increases in the proportion of PPG result in a decrease in the CMT of the copolymer in solution. Suggesting that aggregation and hence, the sol–gel behaviour of poloxamers, is a consequence of the amount of PPG incorporated. The synthesised PCL–PEG–PPG copolymers mirror this behaviour (Figure 4), whereby the concentration of copolymer required to cause gelation, decreases from 20 wt% for PEG_1_PPG_1,_ to 10 wt% for PEG_1_PPG_3_. Gelation at low concentrations is advantageous for ACTs, as it allows for therapeutic amounts of cells to be encapsulated in a minimal amount of copolymer, thus reducing the amount of foreign material introduced in vivo and maximising the efficacy of the treatment. The PCL–PEG–PPG copolymers are advantageous in this respect as they exhibit gelation at concentrations of 10 and 5 wt% (Figure 4f and Figure 4g, respectively). In comparison, the commonly used poloxamer, Pluronics F127, requires concentrations of >15 wt% for gelation to occur [39].

### 3.3. Injectability

The injectability of a thermoresponsive hydrogel formulation is important for determining whether it is suitable for a desired administration route. In ACT, administration may involve direct injection into a tissue and require a syringe needle with a small bore size (e.g., 25–27 G). In contrast, a larger bore size needle (e.g., <19 G) may also be suitable for topical administration at a resection site immediately following surgery. Generally, a formulation can be considered injectable if the force applied to the syringe plunger during administration can be practically applied in a clinical setting. As previously reported [33], this force is dissipated into: (i) initially overcoming the resistance force of the plunger, (ii) imparting kinetic energy to the formulation; and (iii) expelling the formulation through the syringe needle. Previous reports have suggested that if this applied force is <40 N, a formulation can be considered manually injectable [40,41]. To investigate the injectability of the PCL–PEG–PPG copolymers, the injection (into air) of M_0.7_PEG_1_PPG_2_ (10 wt% in PBS), Pluronics F127 (15.5 wt% in PBS), PBS and an empty syringe (control) were profiled using a mechanical tester to generate force versus displacement plots (SI, Figure S1) as previously described [33,42]. For each formulation the plunger-stopper break-loose force (PBF), maximum forces and the dynamic glide force (DGF; average force required to expel the formulation) were determined (Table 2).

The DGF required to inject the M_0.7_PEG_1_PPG_2_ formulation was found to be considerably lower than Pluronics F127, suggesting that a M_0.7_PEG_1_PPG_2_ formulation offers superior injectability. It is important to note that the concentrations of the two formulations were selected as the lowest concentration at which the formulation was a solution at ambient temperature and formed a gel at physiological temperature [43]. This represents an injectable, in-situ gelling formulation for each polymer and demonstrates the favourable injectability of the M_0.7_PEG_1_PPG_2_ formulation for ACT, as injection is possible with gelation occurring thereafter in-situ. Interestingly, the DGF of the M_0.7_PEG_1_PPG_2_ formulation is also slightly lower than both PBS and an empty syringe. Such a reduction in DGF from PBS to M_0.7_PEG_1_PPG_2_ may result from a reduction in friction between the syringe plunger and barrel afforded by the formulation acting as a lubricant. With the exception of Pluronics F127, both the PBF and F_max_ values for each sample indicate that the largest force required was to dislodge the plunger, after which the formulation could flow freely out of the syringe. In contrast, the F_max_ and PBF of Pluronics F127 demonstrate that a greater force was required following the initial dislodgement of the plunger, likely a function of the viscosity of the formulation. These results indicate that the PCL–PEG–PPG copolymers are suitable as injectable thermoresponsive hydrogel formulations at low concentrations, although further ex vivo injectability tests (e.g., using human/animal skin models) are required to determine whether the backpressure incurred when injecting directly into tissue would prelude such direct injections in a clinical setting.

### 3.4. Copolymer Degradation

**Scheme 2:**
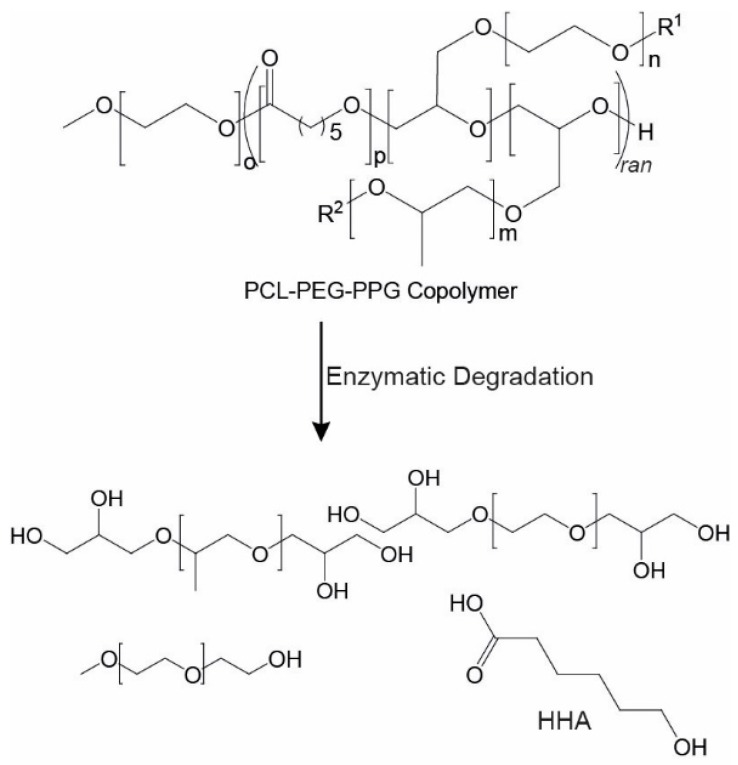
Enzymatic degradation of a PCL–PEG–PPG copolymer showing the potential degradation products if complete degradation was to occur.

In cell therapies, it is desirable to have injectable thermoresponsive gels that are biodegradable or bioerodible to allow their clearance from the body without the need for surgical removal. Furthermore, the degradation products must be non-toxic. One of the major benefits of PCL-based systems, such as the copolymers described, is the presence of hydrolysable and enzymatically degradable ester linkages that allow the materials to be easily degraded in vivo. Previously, several studies have demonstrated that PCL-based materials can be degraded by lipase enzymes [29,44,45], such as those found in the human body. Therefore, the degradation of the copolymer PEG_1_PPG_2_ was studied in the presence of commercially available lipases isolated from *Rhizopus oryzae* (~10 U·mg^-1^) and *Candida Rugosa* (~700 U·mg^-1^). Theoretically, complete cleavage of the PCL ester linkages in the copolymer will result in the formation of 6-hydroxyhexanoic acid (HHA), as well as PEG and PPG components (Scheme 2) [46]. Whereas PEG [47] and PPG [48] are generally regarded as non-toxic and are approved for various biomedical applications, HHA has a high LD_50_ value of ~4.3 g·kg^−1^ [49], which is highly unlikely to be reached from the relatively slow in vivo degradation of PCL-based materials.

**Figure 5:**
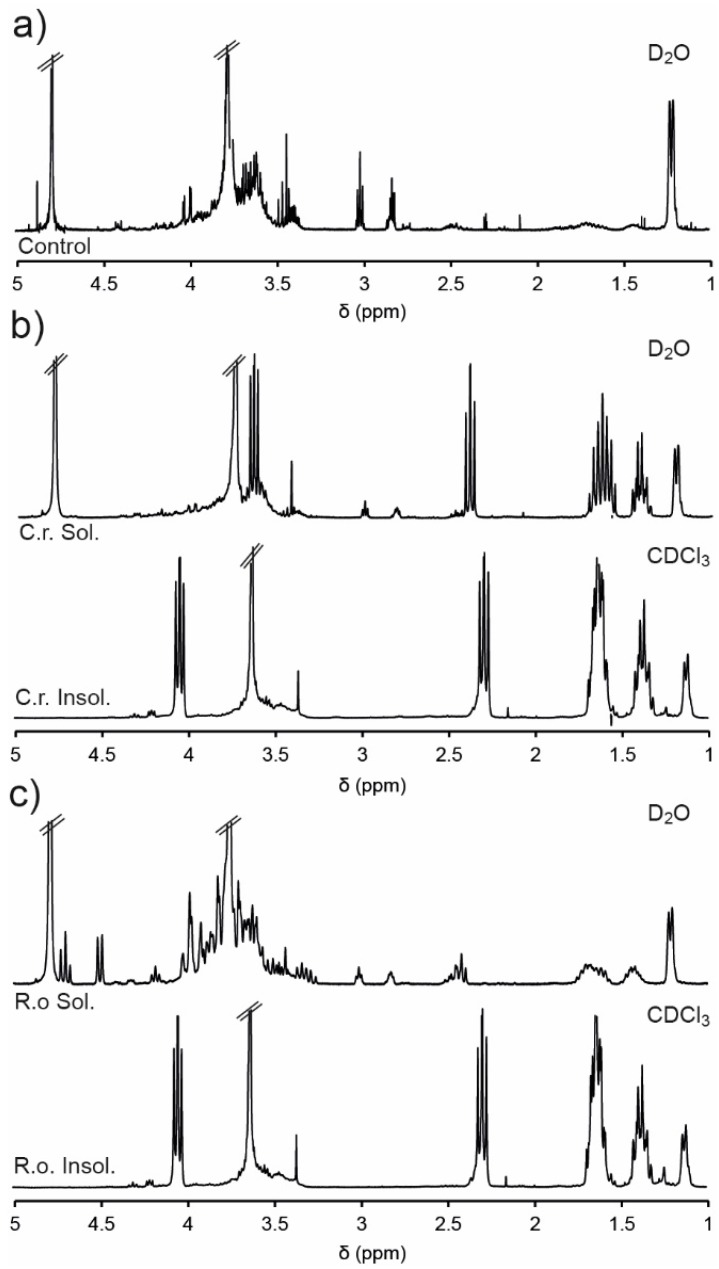
^1^H NMR spectra of (**a**) PEG_1_PPG_2_ in D_2_O (5 wt%) before addition of lipase (control), and D_2_O soluble and insoluble (CDCl_3_) portions following 48 h exposure to (**b**) *Candida rugosa* and (**c**) *Rhizopus oryzae*.

For the degradation studies, PEG_1_PPG_2_ suspensions (5 wt%) were prepared in D_2_O containing the enzymes (10 mg·mL^−1^), and incubated at 37 °C for 48 h. Subsequently, the soluble and insoluble components were separated via centrifugation and analysed via NMR spectroscopic analysis (Figure 5). The D_2_O soluble component of the PEG_1_PPG_2_ copolymer (control) was also analysed by NMR spectroscopic analysis prior to degradation (Figure 5a), which revealed a significant reduction in the intensity of resonances from the PCL segments as compared to the spectrum of PEG_1_PPG_2_ recorded in CDCl_3_ (Figure 1b). This reduction in intensity is likely due to the amphiphilic nature of the copolymer and the restricted segmental mobility phenomenon [50,51]. The hydrophobic nature of the PCL segments causes them to aggregate in water, tightly packing the PCL segments and restricting the mobility of the hydrogen atoms. This restriction in mobility impedes the relaxation of the hydrogen atoms and results in the broadening and weakening of the resonances resulting from the PCL component at ~1.3–1.7, 2.3 and 4.1 ppm (Figure 5a). Conversely, the hydrophilic PEG and PPG segments of the copolymer are solvated, causing strong resonances at 3.6 and 1.1 ppm, respectively.

The NMR spectra of the insoluble portion (recorded in CDCl_3_) isolated from the enzymatic degradation of PEG_1_PPG_2_ (Figure 5b,c) were consistent with the original copolymer (Figure 1b), except for the disappearance of the epoxide resonances (2.6–3.1 ppm), which may result from their hydrolysis or their segregation in the soluble portion. Analysis of the soluble portions revealed evidence of degradation of the copolymer, with resonances at ~2.4 and 3.6 ppm characteristic of the methylene groups adjacent to carbonyl and hydroxyl groups of HHA (Scheme 2). Furthermore, exposure to the enzyme *C. rugosa* appears to result in greater degradation than exposure to *R. oryzae,* as evidenced by the significantly larger HHA resonances. These results are consistent with the greater enzymatic activity of *C. rugosa* compared to *R. oryzae* and indicate that the ester linkages of the copolymers are enzymatically degradable, although the rate of degradation is highly dependent on the particular enzyme.

### 3.5. Cell Encapsulation and Survival

**Figure 6:**
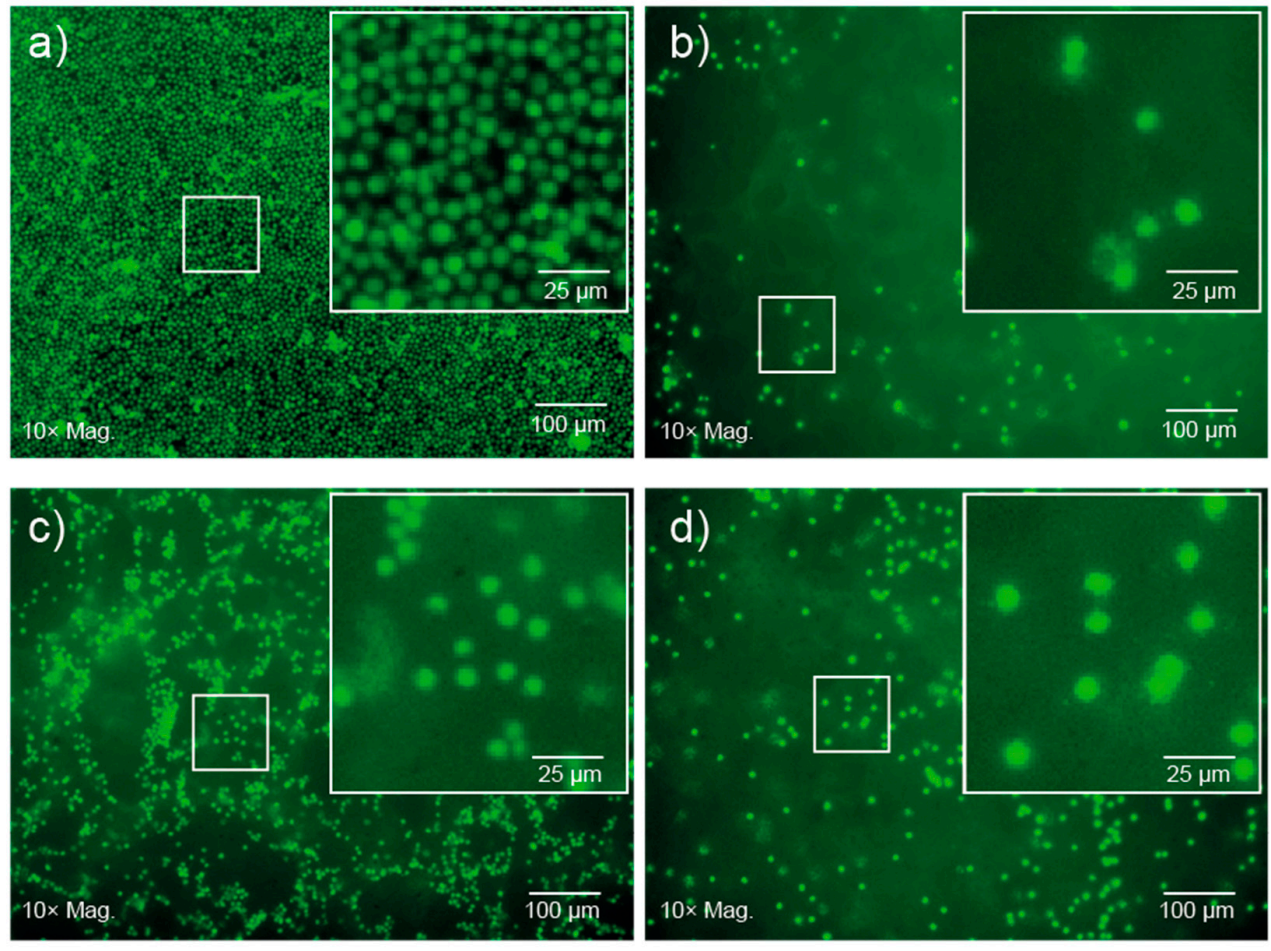
Fluorescence microscopy images of CD4+ T-cells after 24 h (**a**) on well plates at a cell density of 1 × 10^6^ cells·mL^−1^, and encapsulated in 10 wt% PEG_1_PPG_2_ copolymer gels at cell densities of (**b**) 1 × 10^6^ and (**c**) 5 × 10^6^ cells·mL^−1^ and 15 wt% PEG_1_PPG_2_ copolymer gel at a cell density of (**d**) 1 × 10^6^ cells·mL^−1^.

The PCL–PEG–PPG copolymers were developed with the intention that they could be used as injectable, thermoresponsive gels for ACTs. Therefore, preliminary encapsulation studies were conducted with fluorescently labelled CD4+ T-cell lymphocytes in PEG_1_PPG_2_ copolymer gels and qualitative observations of their viability were made via fluorescence microscopy. The lymphocytes were initially mixed with PEG_1_PPG_2_ solutions prepared in cell media at room temperature and then incubated at 37 °C to afford gels with different cell densities. After 24 h, fluorescent imaging revealed no change in the characteristic spherical morphology of the lymphocytes incubated in well-plates or encapsulated within the gel (Figure 6). An apparent reduction in cell number within the focal plane, and diffuse fluorescent signal observed in the images of cells encapsulated in gels (Figure 6b–d) results from the suspension of the cells throughout the gel matrix, and from focusing on the bottom of the gel. Furthermore, no significant changes were observed over 5 d, suggesting that the copolymer gel is not cytotoxic and that the cells are viable after this period of time. This preliminary study suggests that the PCL–PEG–PPG copolymers may be suitable for use as in situ gelling delivery vehicles for ACTs, and further studies are currently underway to comprehensively characterise the interaction between the lymphocytes and the gels, as well as their diffusion and function post-encapsulation. 

## 4. Conclusions

Herein, we report the synthesis of a series of novel thermoresponsive PCL–PEG–PPG copolymers via ring-opening polymerisation of caprolactone and epoxide functionalised PEG and PPG derivatives. ^1^H NMR spectroscopic analysis and GPC revealed that the PEG and PPG components are incorporated into the PCL backbone leading to branched copolymers. DSC revealed that the copolymers displayed melting and crystallisation peaks consistent with PCL, although the enthalpy changes of the transitions were reduced indicating that the PEG and PPG segments inhibit the formation of the PCL crystallites. The extent to which PPG was incorporated into the resultant copolymers had a significant effect upon their gelation and thermoresponsive properties, with gelation occurring at a relatively low concentration (10 wt%) for PEG_1_PPG_3_. This is consistent with the currently proposed mechanisms of gelation of poloxamers, whereby the reduction in solubility of PPG at increased temperatures, drives gelation. PEG_1_PPG_2_ exhibited a physiologically relevant gelation profile and was selected for further experimentation, wherein the enzymatic degradation of the PCL ester linkages to 6-hydroxyhexanoic acid resulted in degradation of the copolymers, thus demonstrating the biodegradability of the PCL–PEG–PPG copolymers. Furthermore, the in vitro encapsulation of CD4+ T-cell lymphocytes in an aqueous PEG_1_PPG_2_ gel, suggested that the copolymer was cytocompatible, with viable cell morphology maintained over 5 d. These data demonstrate that PCL–PEG–PPG copolymers exhibit the relevant physicochemical properties necessary for use as an adoptive cell therapy delivery vehicle, in the form of an injectable, in situ gelling hydrogel, which may enable the delivery of such therapies to solid tumours at target sites that are poorly accessible via conventional intravenous administration, such as the brain. Further studies to quantify cell viability, diffusion and function post-encapsulation are underway to fully elucidate the suitability of these copolymers for in vivo use.

## Figures and Tables

**Table 1:** Initial monomer feed mole ratios used in the synthesis of the copolymers and characterisation of the copolymer composition and molecular weight.

Designation	Monomer Feed Mole Ratio (Repeat Unit Ratio)			Copolymer Mole Ratio (Repeat Unit Ratio) ^a^			Molecular Weight ^b^ (*M*_w_)	Dispersity ^b^ (*Ð*)
	CL	EPEG	EPPG	PCL	PEG	PPG		
**PCL–PEG**	1	0.27 (2.4)		1	0.24 (2.1)		5900	4.9
**PEG_1_PPG_1_**	1	0.13 (1.1)	0.13 (0.9)	1	0.16 (1.4)	0.08 (0.6)	2100	2.8
**PEG_1_PPG_2_**	1	0.08 (0.7)	0.17 (1.2)	1	0.07 (0.6)	0.05 (0.4)	2000	2.8
**PEG_1_PPG_3_**	1	0.06 (0.5)	0.19 (1.4)	1	0.04 (0.3)	0.07 (0.5)	4500	3.1
**M_0.7_PEG_1_PPG_2_**	1	0.08 (0.7)	0.17 (1.2)	1	0.05 (0.5)	0.07 (0.4)	5400	2.9

^a^ Determined from ^1^H NMR spectroscopic analysis resonance integrations. ^b^ Molecular weight characteristics determined by GPC.

**Table 2:** Dynamic glide forces, plunger-stopper break-loose forces and maximum forces determined for M_0.7_PEG_1_PPG_2_ (10 wt% in PBS), Pluronics F127 (15.5 wt% in PBS), PBS, and an empty syringe. Results are expressed as the mean of six determinations ± standard deviation.

Formulation	Plunger-Stopper Break Loose Force (PBF, N)	Maximum Force (F_max_, N)	Dynamic Glide Force (DGF, N)
Empty	20.53 ± 6.75	10.38 ± 4.40	8.53 ± 1.25
PBS	29.84 ± 6.18	13.02 ± 6.54	12.38 ± 0.34
Pluronics F127 (15.5 wt%)	29.19 ± 3.62	39.26 ± 4.44	36.44 ± 1.63
M_0.7_PEG_1_PPG_2_ (10 wt%)	22.07 ± 5.66	10.27 ± 5.59	7.98 ± 0.27

## Supplementary Materials

The following are available online at https://www.mdpi.com/2073-4360/12/2/367/s1. Table S1: Comparison of theoretical and actual epoxide group integrals of the synthesised copolymers; Table S2: Summary of reagent quantities and results of the direct synthesis of the copolymers; Figure S1: Injectability testing of formulations showing force versus displacement plots obtained from the injection of 1 mL of M_0.7_PEG_1_PPG_2_ (10 wt% in PBS), Pluronics F127 (15.5 wt% in PBS), PBS and an empty syringe, via a 1 mL disposable syringe fitted with a 25 G needle.
