# Supplementary material for: Thermoresponsive Poly(ε-Caprolactone)-Poly(Ethylene/Propylene Glycol) Copolymers as Injectable Hydrogels for Cell Therapies
Source: Polymers (Basel). 2020 Feb 7;12(2):367. doi: 10.3390/polym12020367 (PMC7077385; doi:10.3390/polym12020367)
Supplement: Supplementary file 1 [file polymers-12-00367-s001.pdf]

SUPPORTING INFORMATION

Thermoresponsive poly(ε-caprolactone)-poly(ethylene/propylene glycol) copolymers as injectable hydrogels for cell therapies

Kyle Brewer[1,2], Batjargal Gundsambuu[2,3,] Paula Facal Marina[1,2,5], Simon C. Barry[2,3,4], Anton Blencowe[1,2,5,*]

[1] Applied Chemistry and Translational Biomaterials (ACTB) Group, School of Pharmacy and Medical Sciences, University of South Australia, Adelaide, South Australia 5000, Australia.

[2] Cooperative Research Centre for Cell Therapy Manufacturing, University of South Australia, Adelaide, South Australia 5000, Australia.

[3] Molecular Immunology, Robinson Research Institute, University of Adelaide, Adelaide, South Australia 5005, Australia.

[4] Department of Gastroenterology, Women's and Children's Hospital, SA Health, Adelaide, South Australia 5006, Australia.

[5] Future Industries Institute, University of South Australia, Mawson Lakes, South Australia 5095, Australia.

Table S1: Comparison of theoretical and actual epoxide group integrals of the synthesised copolymers.

Copolymer	Theoretical Epoxide (CH) Integral	Actual Epoxide (CH) Integral	Reduction in Epoxide (%)
**PCL-PEG**	0.47	0.22	54
**PEG_1PPG_1**	0.48	0.37	23
**PEG_1PPG_2**	0.25	0.16	36
**PEG_1PPG_3**	0.20	0.13	36
**$M_{0.7}PEG_1PPG_2$**	0.22	0.11	51

Table S2: Summary of reagent quantities and results of the direct synthesis of the copolymers.

Designation	MPEG (g)	EPEG (g)	CL (g)	EPPG (g)	Tin(II) octoate (g)	Solvent Volume (mL)	Weight Average Molecular Weight (M_w)	Number Average Molecular Weight (M_n)	Z-Average Molecular Weight (M_z)	Dispersity (Đ)	Reagent Molar Ratio (EPEG:EPPG)
**PCL-PEG**	2.184	6.116	11.116	7.815	0.172	5	5864	1219	12910	4.844	1:1
**PEG_1PPG_1**	2.184	6.116	11.116	7.815	0.172	5	2067	739	3315	2.796	1:1
**PEG_1PPG_2**	2.194	4.018	11.128	10.407	0.172	5	2001	706	3446	2.834	1:2
**PEG_1PPG_3**	2.184	3.012	11.129	11.712	0.172	5	4472	1454	8730	3.076	1:3
**$M_{0.7}PEG_1PPG_2$**	1.497	4.014	10.963	10.250	0.119	5	5428	1901	10053	2.855	1:2

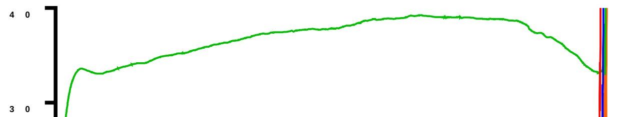

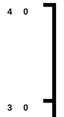

a)

b)

Figure S1. Injectability testing of formulations showing force versus displacement plots obtained from the injection of 1 mL of $M_{0.7}PEG_{1}PPG_{2}$ (10 wt% in PBS), Pluronics F127 (15.5 wt%, in PBS), PBS and an empty syringe, via a 1 mL disposable syringe fitted with a 25G needle:(a) details the entire measurement for each material (n = 6), and (b) highlights the region of the plunger-stopper break loose forces at approximately 0.15 mm.
